# Contribution of Surface-Exposed Loops on the HPV16 Capsid to Antigenic Domains Recognized by Vaccine or Natural Infection Induced Neutralizing Antibodies

**DOI:** 10.1128/spectrum.00779-22

**Published:** 2022-04-27

**Authors:** Anna Godi, Stuti Vaghadia, Clementina Cocuzza, Elizabeth Miller, Simon Beddows

**Affiliations:** a Reference Services Division, UK Health Security Agency (UKHSA), London, United Kingdom; b Department of Surgery and Translational Medicine, University of Milan-Bicocca, Monza, Italy; c Immunisation and Vaccine-Preventable Diseases Division, UKHSA, London, United Kingdom; d Blood Safety, Hepatitis, Sexually Transmitted Infections and HIV Division, UKHSA, London, United Kingdom; University of Arizona

**Keywords:** human papillomavirus, neutralizing antibodies, pseudovirus, surface-exposed loops

## Abstract

Human papillomavirus (HPV) is the causative agent of cervical and other cancers and represents a significant global health burden. HPV vaccines demonstrate excellent efficacy in clinical trials and effectiveness in national immunization programmes against the most prevalent genotype, HPV16. It is unclear whether the greater protection conferred by vaccine-induced antibodies, compared to natural infection antibodies, is due to differences in antibody magnitude and/or specificity. We explore the contribution of the surface-exposed loops of the major capsid protein to antigenic domains recognized by vaccine and natural infection neutralizing antibodies. Chimeric pseudoviruses incorporating individual (BC, DE, EF, FG, HI) or combined (All: BC/DE/EF/FG/HI) loop swaps between the target (HPV16) and control (HPV35) genotypes were generated, purified by ultracentrifugation and characterized by SDS-PAGE and electron microscopy. Neutralizing antibody data were subjected to hierarchical clustering and outcomes modeled on the HPV16 capsomer crystal model. Vaccine antibodies exhibited an FG loop preference followed by the EF and HI loops while natural infection antibodies displayed a more diverse pattern, most frequently against the EF loop followed by BC and FG. Both vaccine and natural infection antibodies demonstrated a clear requirement for multiple loops. Crystal modeling of these neutralizing antibody patterns suggested natural infection antibodies typically target the outer rim of the capsomer while vaccine antibodies target the central ring around the capsomer lumen. Chimeric pseudoviruses are useful tools for probing vaccine and natural infection antibody specificity. These data add to the evidence base for the effectiveness of an important public health intervention.

**IMPORTANCE** The human papillomavirus type 16 (HPV16) major virus coat (capsid) protein is a target for antibodies induced by both natural infection and vaccination. Vaccine-induced immunity is highly protective against HPV16-related infection and disease while natural infection associated immunity significantly less so. For this study, we created chimeric functional pseudoviruses based upon an antigenically distant HPV genotype (HPV35) resistant to HPV16-specific antibodies with inserted capsid surface fragments (external loops) from HPV16. By using these chimeric pseudoviruses in functional neutralization assays we were able to highlight specific and distinct areas on the capsid surface recognized by both natural infection and vaccine induced antibodies. These data improve our understanding of the difference between natural infection and vaccine induced HPV16-specific immunity.

## INTRODUCTION

Human papillomavirus (HPV) is the causative agent of cervical and other anogenital and head and neck cancers and accounts for >600,000 cancer cases globally per annum (1–3). The bivalent (Cervarix) and quadrivalent (Gardasil) HPV vaccines target the most common oncogenic genotypes HPV16 and HPV18, while the nonavalent HPV vaccine (Gardasil9) also targets an additional 5 oncogenic genotypes (HPV31, HPV33, HPV45, HPV52, and HPV58) (4). Quadrivalent and nonavalent vaccines target HPV6 and HPV11 which can cause genital warts. Clinical trial data demonstrate that these vaccines are highly efficacious against vaccine targeted types and vaccine effectiveness studies are beginning to confirm these experimental observations in target populations following introduction of national immunization programmes (5). There are no defined correlates of protection for HPV vaccination. The immune response induced following HPV vaccination is typically monitored by quantitation of the antibody response against each vaccine-incorporated type (6), supported by exploratory data on B and T cell function and *in vivo* protection in an animal model (7). Empirical data on the breadth, magnitude, specificity, and durability of the immune response elicited by the HPV vaccines continue to contribute to improving the evidence base that supports this important public health intervention.

HPV vaccination induces antibody levels orders of magnitude greater than the typical levels of antibodies found in natural infection (8). Little is known about the antibody specificities elicited by natural infection compared to those generated following vaccination although emerging evidence suggests that natural immunity can protect against subsequent reinfection by the same type but much less efficiently than by vaccination (9).

Several studies have attempted to delineate the regions of the L1 capsid that constitute neutralizing antibody domains. Early work using chimeric virus-like particle (VLP) competition demonstrated that some HPV16 natural infection sera could be differentially blocked by DE, FG and/or HI loop mutant VLP (10). Chimeric L1 antigens have been used to map conformational murine monoclonal antibody (MAb) binding sites and loop preferences, including that of the potent neutralizing MAb H16.V5 (11–13). Furthermore, this MAb competes with the binding antibody specificities found in both natural infection and vaccine sera (14, 15) suggesting that the repertoires overlap at least to some extent. H16.V5 has been mapped by cryo-electron microscopy to a domain that includes residues located in multiple surface-exposed loops (BC, DE, FG and HI) (16, 17) and a human MAb, 26D1, whose footprint includes residues also present in the H16.V5 epitope, similarly competes with binding antibodies derived from natural infection and vaccination (18). These data suggest that binding and neutralizing antibodies generated following natural infection and vaccination share at least some specificity and that at least some of this specificity involves the surface-exposed loops. Whether the surface-exposed loops account for the entirety of the neutralizing antibody specificity generated by natural infection or vaccination or whether it is the majority response exemplified by a limited number of neutralizing MAbs is unclear.

Licensed vaccines induce a robust immune memory, even after a single dose, that can be boosted at least 6 years later by a heterologous vaccine dose (19). Evaluation of the antibody responses of individuals seropositive and DNA negative at entry into vaccine trials shows a similar anamnestic response following vaccination suggesting that naturally induced recall memory is also quite robust (20–22). In a seminal study of B cell clones derived following natural infection and after receiving a single vaccine dose, demonstrate differences in both magnitude but also key qualitative differences in the resulting neutralizing antibody capacity (23). Taken together, these data suggest some degree of commonality between specificities of antibodies elicited by natural infection and vaccination but in the latter case are induced to much higher levels.

In this exploratory study, we created chimeric pseudoviruses (PsVs) containing target (HPV16) L1 loops in a background control (HPV35) genotype in an attempt to map and contrast the functional neutralizing antibody specificities present in natural infection sera and vaccine sera to address whether the difference in neutralizing antibody potency between vaccine and natural infection sera was simply due to differences in magnitude or whether vaccination and natural infection also elicited different antibody specificities.

## RESULTS

### Amino acid sequence and biophysical properties of chimeric pseudoviruses.

**FIG 1:**
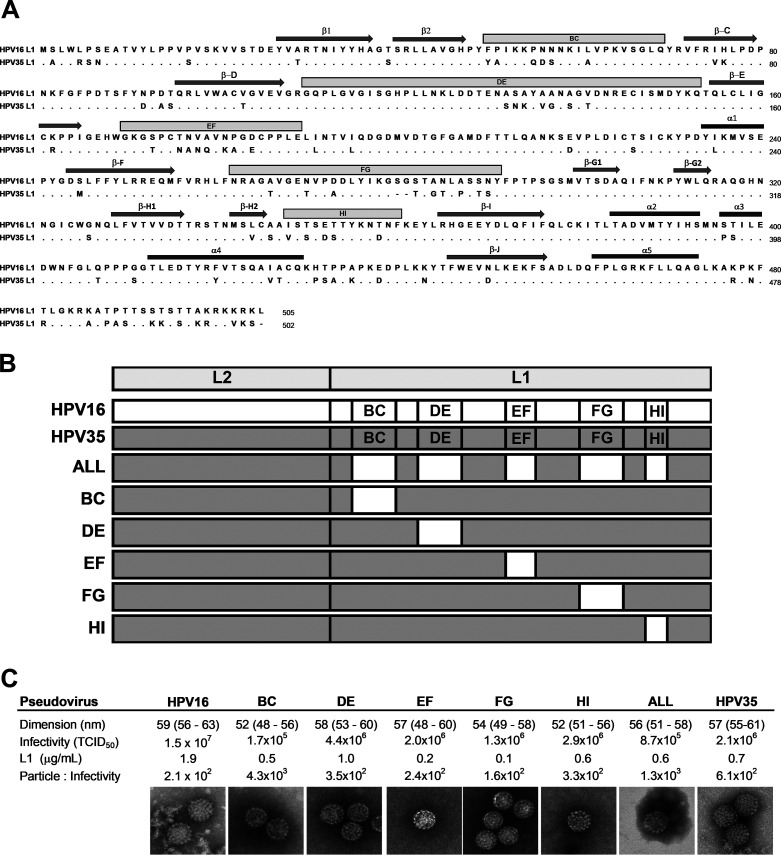
Construction and characterization of HPV35/HPV16 chimeric pseudoviruses. (A) Amino acid alignment of HPV16 (NC_001526) and HPV35 (26) with external surface-exposed loops (BC, DE, EF, FG, and HI) and other features (α-helices and β-sheets) highlighted. Dots depict identity and indels are highlighted as dashes. Numbering is genotype-specific according to the indicated reference for each genotype starting with the second methionine according to convention (https://pave.niaid.nih.gov/). (B) Graphical representation of wild-type (HPV16 and HPV35) and chimeric (HPV35 PsVs containing the indicated HPV16 loop) sequence(s). (C) Pseudovirus preparations characterized for particle dimension (nm median [interquartile range]), infectivity, L1 concentration, and resulting particle-to-infectivity ratio. TCID_50_, 50% tissue culture infectious dose.

The HPV16 (NC_001526) and HPV35 (24) L1 amino acid sequences that formed the basis of the chimeric clones used in this study were aligned and loop positions highlighted (Fig. 1A). Six chimeric PsVs were constructed with each containing HPV35 L1 and L2 genes with a single L1 loop substituted from HPV16 (HPV35_BC_, HPV35_DE_, HPV35_EF_, HPV35_FG_, and HPV35_HI_) or a construct containing all HPV16 loops (HPV35_ALL_) (Fig. 1B). These chimeric PsVs exhibited similar biophysical properties (Fig. 1C).

**FIG 2:**
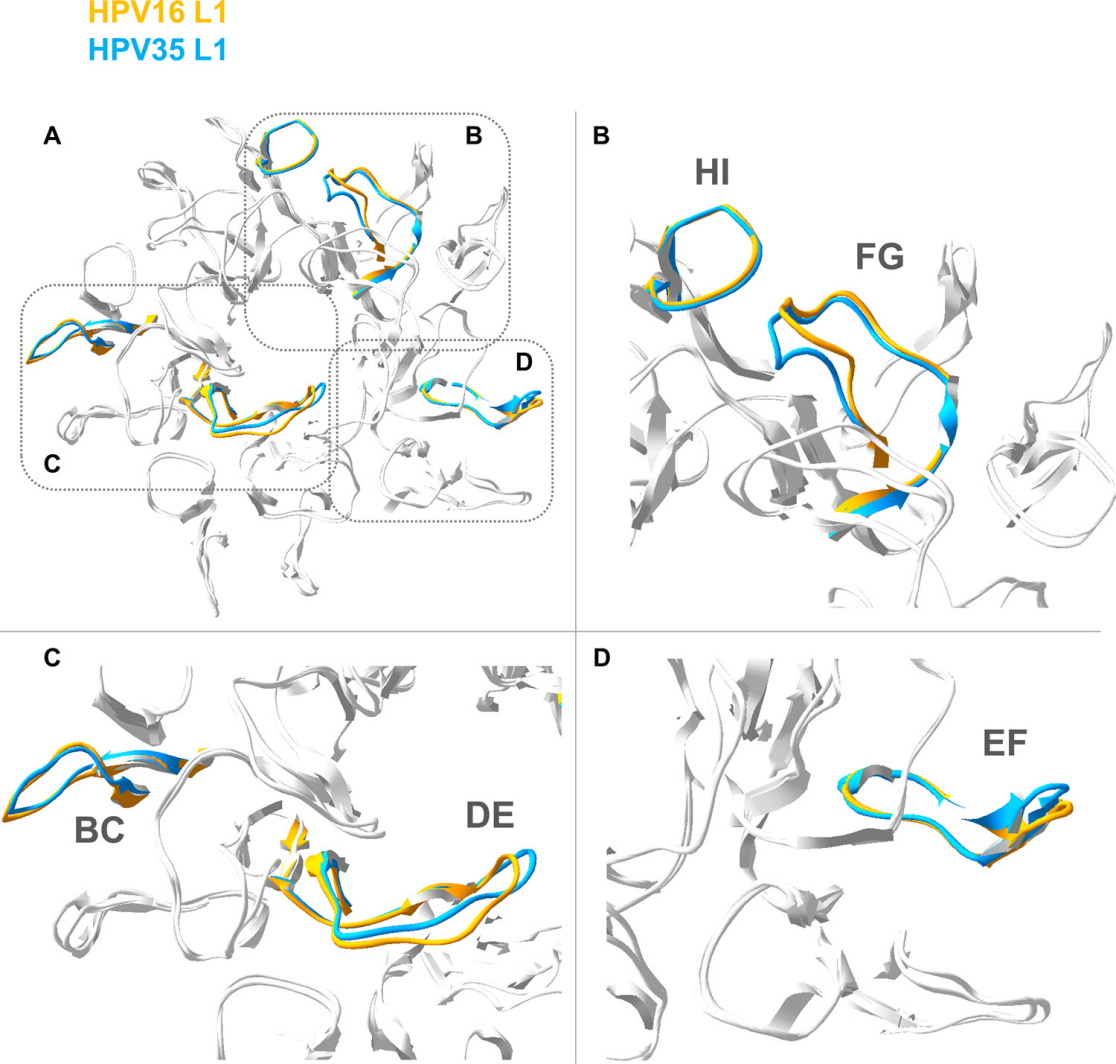
Superimposition model of HPV16 and HPV35 L1 capsomer. (A) Superimposition model of HPV35 (blue; PDB 2R5J) and HPV16 (gold; PDB 2R5H) capsomer crystals to indicate potential structural differences between these genotypes, with individual panels highlighting the (B) FG, HI (C) BC, DE and (D) EF loops. http://doi.org/10.2210/pdb2R5J/pdb; http://doi.org/10.2210/pdb2R5H/pdb.

Superimposition of the HPV16 and HPV35 capsomer crystals highlighted significant structural differences between the capsomers (RMS 0.85 Å), particularly in the external surface exposed loops (Fig. 2). Residues were selected to represent the maximum distance between the two capsomer crystals for each external loop as follows: BC (residue 56, 2.46 Å), DE (residue 137, 3.27 Å), EF (residue 181, 2.27 Å), FG (residue 281, 2.49 Å) and HI (residue 352, 0.79 Å).

### Neutralization of chimeric pseudoviruses by natural infection and vaccine sera.

Serum samples (*n* = 215) obtained from women following a cytological diagnosis of ASCUS or LSIL were tested against both HPV16 and HPV35 PsVs. Samples (*n* = 32) that were positive for HPV16 neutralizing antibodies and negative for HPV35 neutralizing antibodies were further tested against HPV16, HPV35, HPV35_ALL_, HPV35_BC_, HPV35_DE_, HPV35_EF_, HPV35_FG_, and HPV35_HI_ PsVs in parallel. A similarly sized panel of bivalent (*n* = 16) and quadrivalent (*n* = 14) vaccinee sera were tested against these same PsVs in parallel.

**FIG 3:**
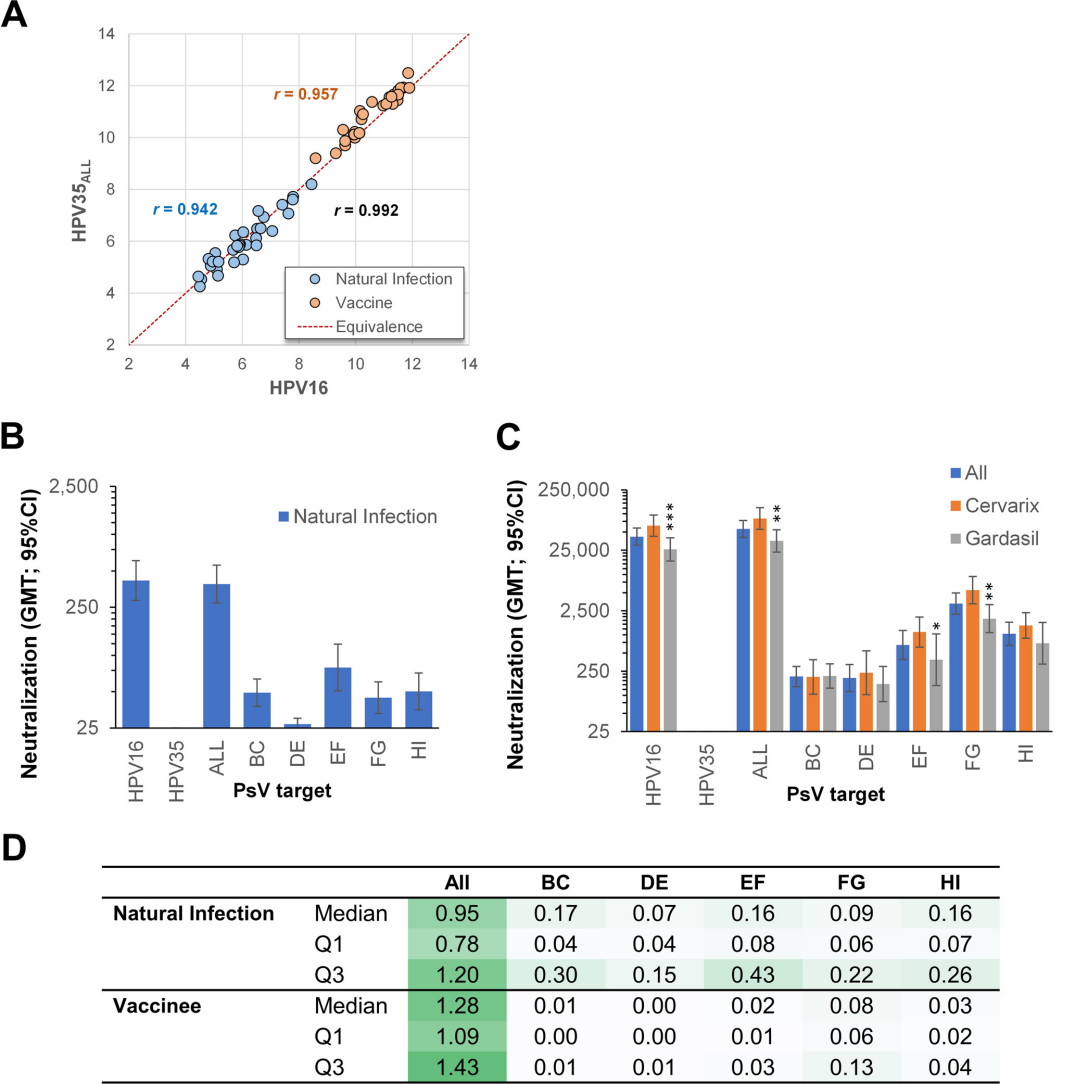
Neutralization of wild-type and chimeric PsVs. Paired natural log titers of natural infection (blue circles with associated Pearson’s *r*) and vaccine (orange circles with associated Pearson’s *r*) sera between the wild-type HPV16 PsV and the chimeric HPV35_ALL_ PsV, with overall Pearson’s *r* depicted in black (A). Geometric mean titers (GMT; 95%CI) are shown for (B) natural infection (*n* = 32) and (C) vaccine (*n* = 30) sera responses against wild-type and individual chimeric PsVs. *, *P* < 0.05; **, *P* < 0.01; ***, *P* < 0.001 were derived using the Kruskal Wallis test for differences between bivalent and quadrivalent vaccine responses. (D) Median (inter-quartile range, IQR; Quartile 1 – Quartile 3) ratio of neutralizing antibody titers against indicated chimeric PsV and HPV16 wild-type PsV for natural infection and vaccine-derived serum.

Sera tested against the HPV16 PsV and the chimeric HPV35 PsV containing all of the HPV16 external loops (HPV35_ALL_) resulted in a median (IQR) ratio of the paired natural log titers of 0.99 (0.95 – 1.03; Pearson’s *r *=* *0.942) and 1.02 (1.01 – 1.03; *r *=* *0.957) for the natural infection (*n* = 32) and vaccinee (*n* = 30) sera, respectively (Fig. 3A). Overall, the neutralizing antibody activity recovered using the chimeric HPV35_ALL_ PsV (median 1.01 [0.99 – 1.03]; *r *=* *0.992; *n* = 62) suggested that almost the entirety of the neutralizing antibody specificity is targeted at the external surface exposed loops.

A substantial minority (6/32; 19%) of natural infection serum samples did not recognize any of the HPV35 single loop chimeric PsVs, despite exhibiting similar titers to the HPV35_ALL_ and HPV16 PsVs, suggesting that the antibody specificities involved required >1 loop to reconstitute relevant neutralizing antibody epitopes. Only 1/32 (3%) of the natural infection serum responses included all loops in the response compared with 24/30 (80%) of vaccinees. The GMT (95%CI) for natural infection serum responses indicate that the majority response was against the EF loop (HPV35_EF_) (Fig. 3B), while for vaccine sera the majority response was against the FG loop (HPV35_FG_) (Fig. 3C). Bivalent vaccine sera exhibited a higher GMT against wild-type HPV16 and the chimeric HPV35_ALL_ PsVs as well as the individual loop-specific PsVs, HPV35_EF_ and HPV35_FG_. Neutralizing antibody titers against individual loops represented a higher proportion of the total HPV16 response for natural infection sera than they did for vaccine sera (Fig. 3D).

**FIG 4:**
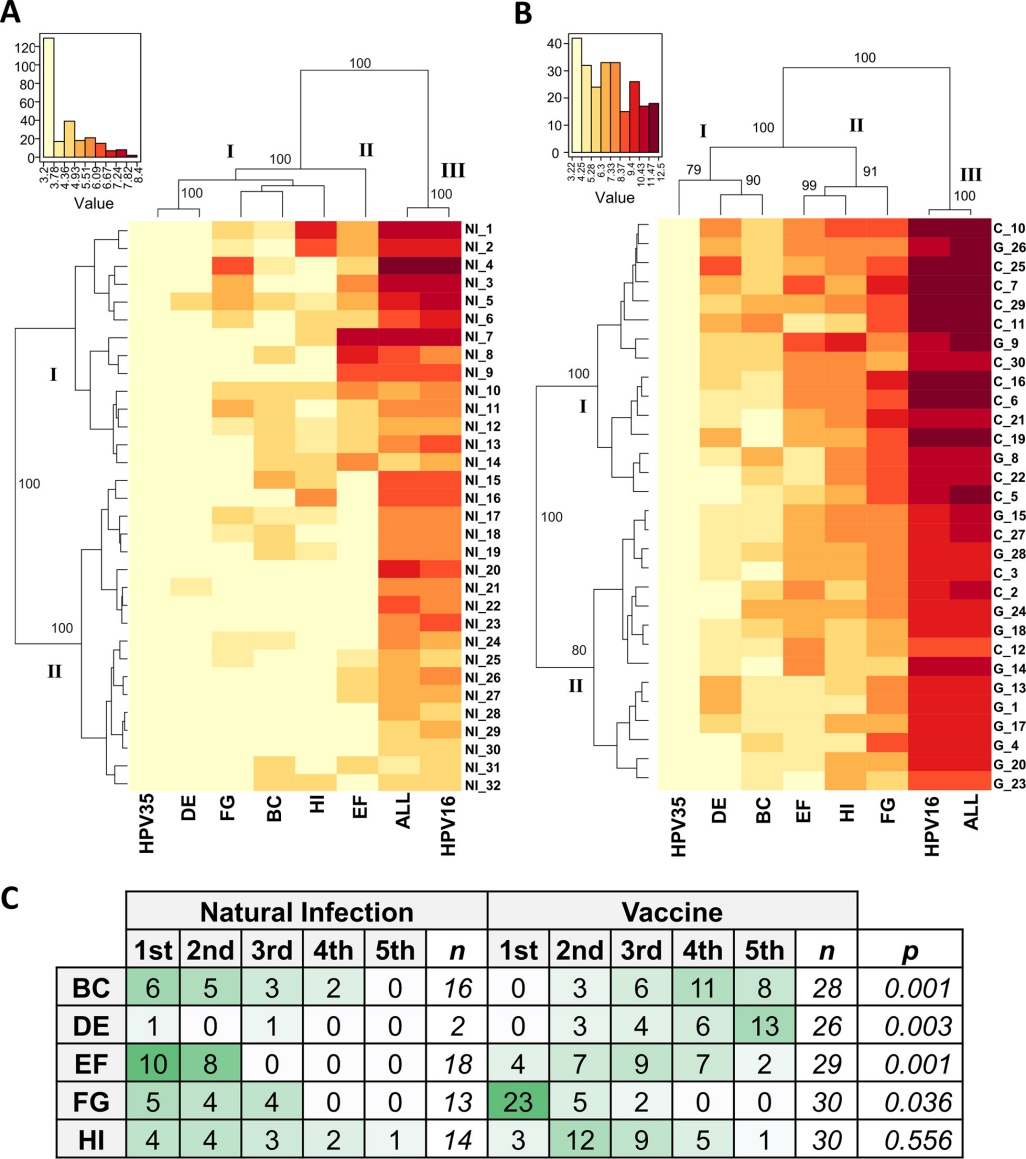
Hierarchical clustering of PsV neutralization data. Hierarchical clustering of the natural log of (A) natural infection and (B) vaccine serum neutralization titers (center, heat map) reordered according to serological (left) and antigen (PsV) (top) dendrograms constructed from the resulting Euclidean distance matrices, with clusters supported by bootstrap values as indicated. (C) distribution of responses for both natural infection and vaccine sera ranked by the magnitude of these responses against individual chimeric HPV35 PsV. Chi^2^ was used to test for differences in sample distribution for each loop between the natural infection and vaccine sources.

To explore these specificities further, natural infection (Fig. 4A) and vaccine (Fig. 4B) neutralization data were subjected to hierarchical clustering (http://www.hiv.lanl.gov/content/sequence/HEATMAP/heatmap.html). The HPV35_ALL_ chimeric PsV clustered together with the HPV16 reference PsV in pseudoviral dendrograms representing both the natural infection and vaccine sera responses. Loop-specific chimeric PsVs were clustered differently, however, due to their reactivity with either natural infection or vaccine sera. For the natural infection sera, the HPV35_EF_ PsV clustered separately from chimeric HPV35 PsVs containing the BC, FG or HI loops and the DE loop. For the vaccine sera, HPV35_FG_ PsV clustered apart from the chimeric HPV35 PsVs containing the EF or HI loops which clustered apart from those chimeric PsVs containing the BC or DE loops. For the vaccine sera dendrogram there was a higher proportion of bivalent vaccine sera associated with Cluster I compared to Cluster II (Chi^2^, *P* = 0.003), but overall both vaccines showed similar antibody specificities that were different from those specificities elicited by natural infection.

The neutralizing antibody titers for each serum against each single loop chimeric PsV were ranked according to their magnitude and the distribution of these rankings was evaluated (Fig. 4C). For example, 16/32 (50%) of natural infection sera responded against the BC loop and these responses tended to be the highest titers against any of the chimeric PsVs for each serum placing this loop toward the highest rankings for natural infection sera while being of a lower relative priority in vaccine sera (*P* = 0.001). Similarly, the EF loop exhibited the highest ranking for natural infection sera while for vaccinee sera its importance tended toward the middle ranks (*P* = 0.001). Naturally infected individuals rarely (2/26; 8%) neutralized the HPV35_DE_ PsV in contract to the majority of vaccinees (26/30; 87%; *P* = 0.003) suggesting that the DE loop plays a minor role in natural infection antibody specificity compared to that of vaccinees. Overall, these data support a differential distribution of the importance of each loop between natural infection and vaccine sera.

**FIG 5:**
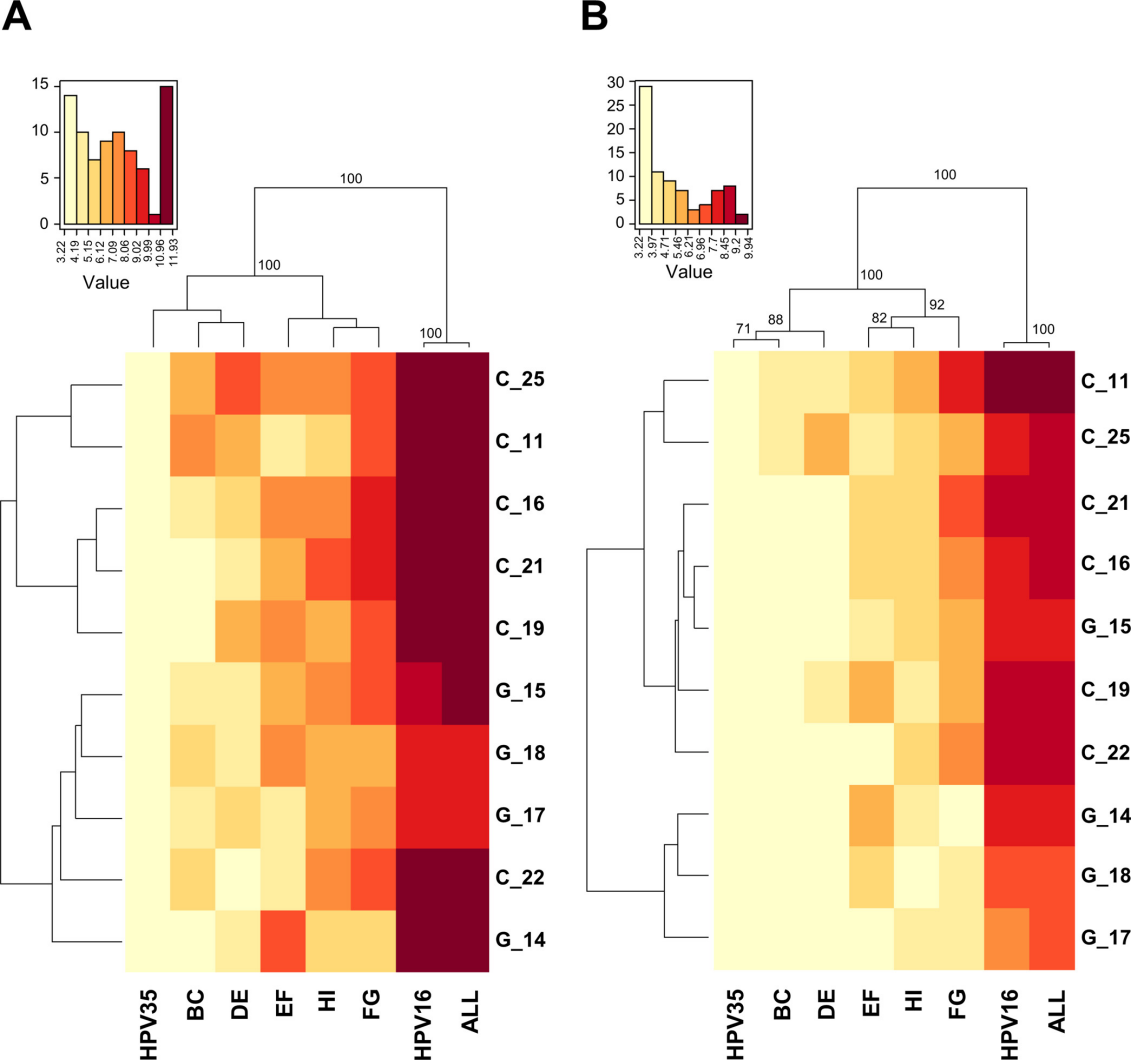
Contribution of the magnitude of antibody response to hierarchical clustering pattern. Hierarchical clustering of (A) vaccine sera and (B) admixed diluted vaccine sera natural log PsV neutralization titers (center, heat map) reordered according to serological (left) and antigen (PsV) (top) dendrograms constructed from the resulting Euclidean distance matrices, with clusters supported by bootstrap values as indicated.

One potential confounding factor is the higher magnitude of the vaccinee neutralizing antibody response compared to the relatively low natural infection antibody response. To address this, lower titer vaccine sera were simulated by admixing individual vaccine sera (*n* = 10) into HPV negative plasma (25) prior to titration against HPV16, HPV35 and each chimeric PsV and tested alongside the untreated sera in parallel. The admixed sera generated a median neutralizing antibody titer 15 (IQR 13 – 16) fold lower than the original untreated sample against the HPV16 PsV but displayed a similar specificity profile against the chimeric PsVs. Thus, hierarchical clustering generated the following PsV antigen clusters: HPV35/HPV35_DE_/HPV35_BC_, HPV35_EF_/HPV35_HI_/HPV35_FG_ and HPV35_ALL_/HPV16 which was identical to the profile displayed by the undiluted sera in this subset (Fig. 5) and the full panel of vaccine sera (Fig. 4B).

**FIG 6:**
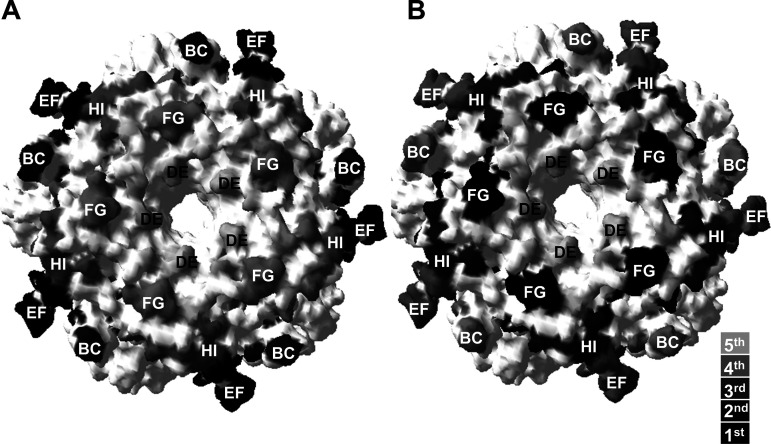
Proposed neutralizing antibody domains on HPV16 L1. Surface-filled HPV16 capsomer model (PDB 2R5H) highlighting residues within external loops that differ between HPV16 and HPV35 (PDB 2R5J). Shading represents the rank order of serum responses against individual chimeric PsV for (A) natural infection and (B) vaccine sera.

The relative importance of the loops in the natural infection and vaccine neutralizing antibody responses based upon the ranking of these responses (Fig. 4C) were transposed onto the HPV16 capsomer crystal map (Fig. 6) in order to contrast both the spatial importance of individual loops in the antibody responses but also to highlight and therefore implicate the specific amino acid residues that differ between the HPV16 and HPV35 sequences in these loops (Fig. 1A). These neutralizing antibody patterns suggested natural infection antibodies typically target the outer rim of the capsomer (exemplified by the BC, EF and FG loops) while vaccine antibodies primarily target the central ring around the capsomer lumen (exemplified by the DE, FG, HI loops).

## DISCUSSION

In this study, we explored the HPV16-specific neutralizing antibody specificities of vaccine and natural infection sera using chimeric HPV35 PsVs expressing surface-exposed loops derived from the HPV16 L1 major capsid protein. The HPV35 genotype is genetically close to HPV16 but antigenically distant as it is largely insensitive to vaccine antibodies (24, 26, 27). Chimeric PsVs (28) or VLPs (10, 13, 29) containing individual loops from another genotype are useful tools that have been used by ourselves and others to explore the antibody specificity of MAbs, natural infection and vaccine sera.

The chimeric HPV35 PsV expressing all the external surface-exposed loops of HPV16 (HPV35_ALL_) yielded a similar neutralizing antibody response to that of the wild-type HPV16 PsV, suggesting that the majority of vaccine and natural infection antibodies target the external loops. These data are consistent with and extend observations that the external surface-exposed loops are the most variable portions of the L1 capsid (30, 31) and are the target for most HPV16 type-specific neutralizing antibodies mapped thus far (17).

We next explored the role of individual loops in the neutralizing antibody response for each serum by using chimeric HPV35 PsVs expressing each individual external HPV16 loop (BC, DE, EF, FG and HI). This approach suggested that the reconstitution of the total antibody response required the appropriate presentation of antigenic domains comprising residues from multiple external loops. This was clearly the case for all vaccine sera and most, but not all, natural infection sera. This is in keeping with published data on the mapping of some neutralizing HPV16 L1 MAbs, which highlight the importance of individual residues located in multiple external loops (13, 16, 17). The murine (H16.V5) (14, 15) and human (26D1) (18) neutralizing MAbs recognize overlapping epitopes that include residues located in multiple loops (DE, FG and HI) and can compete with both vaccine or natural infection antibodies for their binding sites (16, 17).

In this study, there were notable differences in the magnitude, ranking and distribution of vaccine and natural infection antibody responses against these HPV35 chimeric PsVs. Hierarchical clustering of these data suggested that the neutralizing antibody profiles of vaccine and natural infection sera were qualitatively different as these differences were not simply dependent on the magnitude of the response. Overall, these data suggest that natural infection antibodies preferentially recognized domains that include residues located toward the edge of the capsomer, including residues in the BC, EF and FG loops, while vaccine antibodies displayed a preference toward residues within the more centrally located FG and HI loops.

As expected (26), bivalent vaccine sera tended to have higher magnitude antibody titers against wild-type HPV16 PsV compared to quadrivalent vaccine titers. Antibody responses against the chimeric PsVs, however, suggested that bivalent and quadrivalent vaccines elicit similar but nevertheless distinct antibody specificities.

There are several shortcomings intrinsic to the approach taken in this study that should be balanced with the interpretation of these findings. Chimeric PsVs are artefactual constructs created to represent specific empirical manipulations. How appropriately these manipulations are represented is unclear although efforts were made to assess PsV integrity, such as particle formation and the normalization of infectivity, and use genetically similar templates, other more subtle aberrant changes may have been introduced that may have affected the antigenic properties of individual chimeric PsVs. That said, the primary outcome from this study was a comparison of the neutralizing antibody specificity patterns derived using natural infection and vaccine serum across the range of chimeric PsVs used rather than a direct comparison of individual chimeric PsVs, which should mitigate much of this concern. In addition, although the introduction of a single foreign loop confers specific amino acid sequence changes related only to that introduction, the structural impact of these substituted residues on other extra-loop regions cannot be ruled out. Chimeric antigens are useful to highlight the impact of specific substituted residues on a function or property of an antigen but cannot address the contribution of residues that are the same in both parental sequences. We have previously shown that chimeric PsVs incorporating homologous or heterologous lineage specific L2 proteins were neutralized similarly by vaccine sera, natural infection sera, MAbs and animal antisera (32–34). We have also tested vaccine and natural infection sera in binding assays against L1 and L1L2 antigens and found no significant differences (unpublished data). However, it is conceivable that there could be, perhaps subtle, differences in the antigenicity of the L1 major capsid protein arising from the incorporation of L2 and that this leads to a differential immunogenicity between L1-based licensed vaccines and native virions.

Nevertheless, the use of chimeric antigens, particularly those based upon functional antigens (such as PsVs), have proved to be useful tools to delineate antibody specificity in a range of settings (10, 13, 28, 29). The observations described here suggest that natural infection and vaccination derived antibody specificities are qualitatively different, based upon apparent loop preference of the antibody responses. However, this distinction is based upon a single target antigen, HPV16 in this case, and may not be wholly applicable to antigens of other genotypes which may display different antigenic profiles (35). Finally, the natural infection sera were derived from a single cohort and may not be representative of all HPV16 natural infection immune responses.

In summary, chimeric PsVs were used as antigenic tools in an exploratory study to differentiate the functional antibody specificities elicited by natural infection compared to bivalent or quadrivalent vaccination. These data support a difference in both the magnitude and specificity of natural infection antibodies compared to vaccine induced antibodies and add to the evidence base for the effectiveness of an important public health intervention.

## MATERIALS AND METHODS

### Study samples.

Serum samples from 12 to 15-year-old girls randomized to receive three doses of Cervarix or Gardasil as part of a phase IV clinical trial comparing HPV vaccine immunogenicity (www.clinicaltrials.gov: NCT00956553; REC number 09/H0720/25) (26) were used for this study. Serum samples were obtained from women (Gynaecology Outpatients Clinic, San Gerardo Hospital, Monza, Italy; ethics committee reference 08/UNIMIB-HPA/HPV1; No. 1191) following a cytological diagnosis of atypical squamous cells of undetermined significance (ASCUS) or low-grade squamous intraepithelial lesion (LSIL). Samples from this cohort of women were used to provide an estimate of the typical type-specific antibody specificities generated during natural infection.

### L1L2 pseudovirus construction.

Bicistronic psheLL vectors containing codon-optimized L1 and L2 genes for the expression of wild-type HPV16 and HPV35 PsVs have been described previously (24). Chimeric HPV35 L1 genes containing the individual BC (HPV35_BC_), DE (HPV35_DE_), EF (HPV35_EF_), FG (HPV35_FG_) or HI (HPV35_HI_) loop sequences of HPV16 and a construct comprising all of these loop sequences (HPV35_ALL_) were synthesized (GeneArt; Thermo Fisher Scientific) with additional site-directed mutagenesis (QuikChange site-directed mutagenesis kit, Agilent Technologies) as required and cloned into the psheLL HPV35 L2-containing vector. All constructs were confirmed by Sanger sequencing. PsVs were generated by transfection of 293TT cells and purified by ultracentrifugation on an iodixanol (Sigma-Aldrich) gradient following the alternative protocol as previously described (24). Particle formation and size were determined by electron microscopic analysis of negatively stained particles. 10 PsV particles from each preparation were measured (nm) and the median diameter and interquartile range (IQR) calculated. The equivalent of a 50% tissue culture infectious dose (TCID_50_) was estimated for each PsV preparation using the Spearman-Karber method. The L1 concentrations of PsV stocks were determined by semiquantitative Western blot using the CamVir-1 antibody (Abcam) and particle-to-infectivity ratios were determined as previously described (32).

### Neutralization assay.

The PsV neutralization assay was performed as previously described (24). A standardized input of 300 TCID_50_ was used for all PsVs. The neutralizing antibody titer was assigned as the reciprocal of the serum dilution that resulted in an 80% reduction in the luciferase signal compared to control wells (PsV and cells only), estimated by interpolation. For analysis purposes, serum titers less than the limit of detection (LOD, 50) were assigned a censored value of 25. For each serum, a single serial dilution series was tested against all relevant wild-type and chimeric PsVs in parallel.

For quality assurance purposes, a subset of natural infection (*n* = 18) and vaccine (*n* = 10) sera were retested against the HPV16, HPV35, HPV35_BC_, HPV35_DE_, HPV35_EF_, HPV35_FG_, and HPV35_HI_ PsVs resulting in a median ratio for the initial and repeated natural log titers of 1.00 (IQR 1.00 – 1.06; *r^2^* = 0.908; *n* = 196 data pairs).

### Capsomer crystal modeling.

DeepView Swiss-Pdb viewer v4.0 (http://swissmodel.expasy.org/) was used to perform pairwise L1 pentameric crystal comparisons between HPV16 (Protein Data Bank [PDB] code: 2R5H) and HPV35 (PDB code: 2R5J) by superimposition. The superimposition of L1 pentameric structures was supported by a root mean square deviation (RMS) value, which represents the average Å distance between corresponding atoms in the two models.

### Statistical analysis.

Pearson’s *r* was used to compare the responses against wild-type HPV16 and the chimeric HPV35_ALL_ PsV. Kruskal Wallis test was used to compare the bivalent and quadrivalent vaccine responses against wild-type and chimeric PsVs. Chi^2^ test was used to compare the ranked order of the antibody responses of individual sera against the individual chimeric PsVs. All statistical tests were performed using the statistical package Stata 15 (StataCorp, College Station, TX).
